# A retrospective study of the prevalence of bovine fasciolosis at major abattoirs in Botswana

**DOI:** 10.4102/ojvr.v83i1.1015

**Published:** 2016-06-13

**Authors:** M. Ernest Mochankana, Ian D. Robertson

**Affiliations:** 1 Department of Animal Science, Botswana College of Agriculture, Botswana; 2 College of Veterinary Medicine, Murdoch University, Australia

## Abstract

A retrospective study covering a period of ten years (2001–2010) was conducted using postmortem meat inspection records of the Department of Veterinary Services in Gaborone to determine the prevalence of bovine fasciolosis in Botswana. Meat inspection records of monthly and annual returns from the two main export abattoirs in the country were examined, as well as the data collected on the total number of cattle slaughtered and the number of livers condemned due to *Fasciola gigantica* infection. Only 1250 of the approximately 1.4 million cattle slaughtered were infected with *F. gigantica* (0.09%, 95% confidence intervals [CI] 0.0% – 0.3%). No distinct seasonal pattern was observed in condemnation rates of livers. However, the pattern of distribution of fasciolosis was higher (but not significant) in cattle that originated from areas with high rainfall and more permanent water bodies than those from relatively low rainfall areas with a transitory water system. It is recommended that a longitudinal survey should be carried out at the abattoirs and farms to determine the prevalence of the disease in cattle of different ages, sex and breed as well as the place of origin in the country. The present study indicated that the prevalence of fasciolosis in cattle is low and the disease is therefore of less significance in Botswana than other African countries for which information is available.

## Introduction

Fasciolosis, also known as liver fluke disease, is a hepatic parasitic infection caused by trematodes of the genus *Fasciola* which affects numerous mammalian species, mainly ruminants (Gajewska, Smaga-Kozlowska & Wisniewski 2005) in most countries of the world. The two most important species are *Fasciola hepatica* and *Fasciola gigantica*. These liver flukes are significant pathogens of domestic animals, in particular cattle and sheep (Jones, Hunt & King 2006). As *Fasciola* are haematophagous, their infection usually results in anaemia (Phiri, Phiri & Harrison 2007) and can cause a high proportion of mortalities, especially in small ruminants and calves (Mungube *et al*. 2006).

Lymnaeid snails, the intermediate hosts of *Fasciola* spp., play a vital role in the epidemiology and distribution of fasciolosis (Coelho & Lima 2003; Pfukenyi & Mukaratirwa 2004). Therefore, fasciolosis is prevalent in areas where climatic conditions are favourable for the survival of the snail intermediate host, such as marshland pastures (McGavin & Zachary 2007).

In most African countries, the prevalence of fasciolosis in ruminants has been determined through slaughterhouse surveys (Mungube *et al*. 2006; Mwabonimana *et al*. 2009; Phiri *et al*. 2005b). Information gathered on animals slaughtered at an abattoir can be a convenient and relatively inexpensive source of information (Roberts & Suhardono 1996), with the condemnation rates providing a useful guide to the prevalence of the subacute, mild or chronic forms of the disease in the regions served by the abattoirs (Pfukenyi & Mukaratirwa 2004). The data can be used to determine trends in prevalence and significance of the disease, especially where the reporting system is reliable (Roberts & Suhardono 1996).

In Botswana, both the traditional (cattle post) grazing areas and commercial ranches (large fenced farms) send their animals for slaughter to various abattoirs throughout the country. The cattle are usually brought to these abattoirs on foot or by road or rail transport. Meat inspection in the abattoirs is carried out independent of the owners.

The prevalence of a number of diseases, notably bovine cysticercosis, has been reported from data collected at abattoirs in the country. However, no recorded studies have been carried out to determine the prevalence of fasciolosis in cattle using abattoir records. The objective of the present study was to determine the prevalence of *Fasciola gigantica* infections in slaughtered cattle based on the records from the two main export abattoirs in Botswana for the period 2001–2010.

## Materials and methods

### Sampling method

**FIGURE 1:**
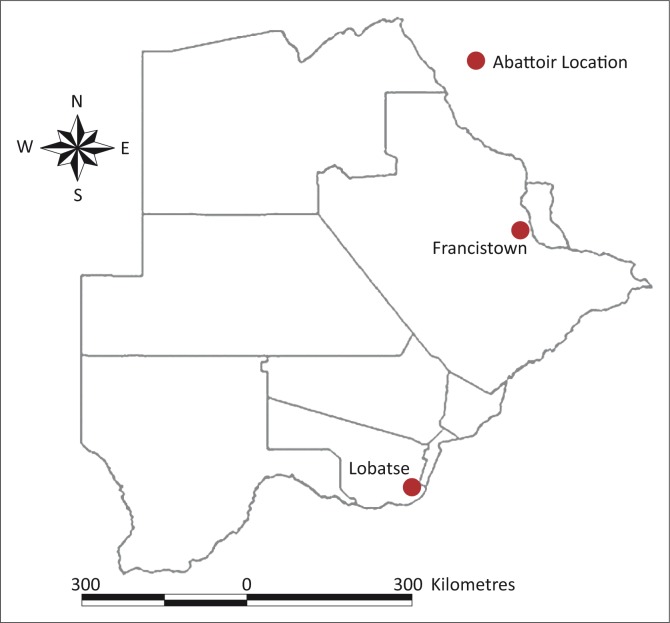
Map showing location of the two sampled export abattoirs in Botswana.

The study involved the retrieval and analysis of meat inspection data from two major Botswana Meat Commission (BMC) export abattoirs at Lobatse and Francistown (Figure 1). These are the largest abattoirs in Botswana, with wide catchment areas, and are located in the southern and northern parts of the country, respectively.

The BMC abattoirs are parastatal organisations jointly owned by the Government of Botswana and private companies, whereas smaller municipal abattoirs are owned by the local governments in the districts. The selected abattoirs covered all regions of the country, and consequently a range of climatic conditions, and serviced both the communal and commercial beef sector farmers, with concurrent different systems of cattle management. Data for the ten-year period, from 2001 to 2010, were collected to estimate the prevalence of fasciolosis in cattle in Botswana.

Meat inspection is performed by certified meat inspectors in accordance with the standards of the *Livestock and Meat Industries Act* of 2007 of the Republic of Botswana, under the supervision of the Chief Veterinary Officer in the Department of Veterinary Services of the Ministry of Agriculture.

### Data collection and analysis

The data were obtained from the two main export abattoirs for the period from January 2001 to December 2010. Records of monthly and annual returns from the abattoirs were scrutinised with regard to the number of cattle slaughtered and the corresponding number of livers condemned as a result of infection with *F. gigantica*. The prevalence of fasciolosis was calculated as the number of cattle infected with *Fasciola* expressed as a percentage of the total number of cattle slaughtered, and was calculated annually for each abattoir. The overall prevalence for the 10-year period (2001–2010) was also determined for each abattoir, together with their 95% CI.

Data obtained for the prevalence of bovine fasciolosis were entered, validated and calculated in Microsoft Excel 2007 spreadsheet, and later transferred into IBM Statistics Programme for Social Sciences (IBM SPSS) version 21.0 for Windows (IBM Corporation, New York, USA) for statistical analysis.

The Mann–Whitney U test was used for the analysis. Descriptive statistics were used to calculate the mean and the 95% CI for the different subgroups.

## Results

The results of the number of cattle slaughtered and condemned livers in each of the two abattoirs over the 10-year period are displayed in Table 1. Out of the 929 937 and 464 784 cattle slaughtered at Lobatse and Francistown abattoirs, 17 and 1232 livers were condemned, respectively. In total, only 1250 livers (0.09%; 95% CI 0.08% – 0.09%) out of almost 1.4 million livers examined were condemned because of *F. gigantica* infestation.

**TABLE 1:** The number of cattle slaughtered and livers condemned due to *Fasciola gigantica* infections at two export abattoirs in Botswana.

Abattoir	Number cattle slaughtered at abattoir	Year										Total for 10 years

		2001	2002	2003	2004	2005	2006	2007	2008	2009	2010		
Lobatse	Number cattle slaughtered	92 466	84 739	100 195	75 698	74 107	95 902	114 018	76 602	91 761	124 449	929 937
	Number livers condemned	7	4	0	0	0	7	0	0	0	0	18
	Percentage infected	0.008	0.005	0	0	0	0.007	0	0	0	0	0.002
Francistown	Number cattle slaughtered	71 200	28 825	53 301	54 022	44 093	41 452	57 211	36 690	43 525	34 465	464 784
	Number livers condemned	9	13	0	0	17	369	773	35	13	3	1232
	Percentage infected	0.013	0.045	0	0	0.039	0.89	1.351	0.095	0.03	0.009	0.265
Annual totals	Total cattle slaughtered	163 666	113 564	153 496	129 720	118 200	137 354	171 229	113 292	135 286	158 914	1 394 721
	Total livers condemned	16	17	0	0	17	376	773	35	13	3	1250
	Percentage infected	0.01	0.015	0	0	0.014	0.274	0.451	0.031	0.010	0.002	0.090

**FIGURE 2:**
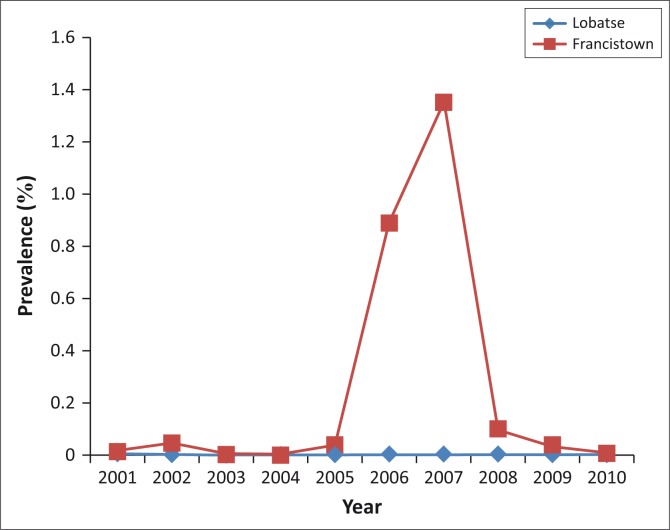
Ten-year (2001–2010) annual trend of the prevalence of fasciolosis in cattle at two export abattoirs in southern and northern Botswana.

A 10-year summary to compare the trend in prevalence of bovine fasciolosis between the two export abattoirs is presented in Figure 2. The annual prevalence at the Francistown abattoir varied between 0% and 1.35%, and at the Lobatse abattoir between 0% and 0.01% (Figure 2 and Table 1). The overall 10-year prevalence of fasciolosis in cattle between the two abattoirs varied between 0.002% at Lobatse and 0.265% at Francistown. The prevalence at Francistown abattoir (0.27%; 95% CI 0.25–0.28) was significantly higher than that at Lobatse abattoir (0.002%; 95% CI 0.00–0.00) (*p* [0.003] < 0.05).

The supply of cattle for Lobatse abattoir, at which prevalence is lower, originates from the southern half of the country, mainly from the two major cattle-producing areas of Gantsi and Kgalagadi districts, in the south-west and western parts of the country, but also from southern, south-east, Kgatleng and Kweneng districts. In contrast, the Francistown abattoir is supplied by the central and north-east districts. The southern and western parts of the country receive less rainfall compared to the central and north-eastern areas of the country. There are also more and larger rivers in the central and north-eastern parts of Botswana.

## Discussion

The findings from this study demonstrate that although fasciolosis was present in cattle slaughtered at the two main export abattoirs in Botswana, the prevalence was low. This is the first systematic record of the prevalence of bovine fasciolosis in the country, and it provides evidence for the need for more extensive epidemiological investigations to be undertaken in different regions of the country. The mean overall prevalence (0.1%) of fasciolosis in cattle slaughtered at the two main export abattoirs in Botswana was much lower than that reported from other countries in sub-Saharan Africa. Similar abattoir studies in Zimbabwe (Pfukenyi & Mukaratirwa 2004), Kenya (Kithuka *et al*. 2002; Mungube *et al*. 2006) and Tanzania (Mellau, Nonga & Karimuribo 2010; Nonga *et al*. 2009) reported a higher prevalence of 37.1%, 8%, 26%, 16.5% and 8.6%, respectively. The present findings rank among the lowest in Africa, and suggest that liver fluke infection in slaughtered cattle in Botswana is not of clinical economic importance.

The abnormal surge in condemnations of *Fasciola*-infected livers in 2006 (*n* = 269), and even more striking in 2007 (*n* = 773), even though the latter was a drought year in Botswana, could be attributed to the high rainfall received by the country in 2005 and the above average rainfall in 2006, which led to higher infections due to increased contact between cattle and contaminated pastures. The most plausible explanation, however, could be that the actual drought that prevailed in 2007 could have led to increased supply (sale) of cattle to the Francistown abattoir as a management measure by farmers, to mitigate the high costs associated with the purchase of supplementary feeds during drought. This highlights the potential bias of extrapolating results from abattoirs to the general population. The limitations associated with abattoir studies include the bias of age and low prevalence of clinical disease (Robertson & Blackmore 1985), as most often the relatively younger and healthier animals are sent for slaughter.

The difference in prevalence of fasciolosis might be attributable to ecological and climatic variations in different areas as well as animal husbandry practices, which differ between countries. This variation could also be due to different internal parasite control between countries. In Botswana, the commonly used veterinary drugs are sold to farmers at subsidised prices from the livestock advisory centres (LACs) to improve livestock production. Therefore, farmers regularly use anthelmintics, and although they use blanket treatment or indiscriminate dosing of their animals, they indirectly could be treating for fasciolosis, as well. One commonly used anthelmintic is albendazole (Valbazen^®^), which is a broad-spectrum drug with flukicidal activity, as well as killing adult trematodes, nematodes and cestodes. Triclabendazole, which is a highly effective flukicide against both adult and juvenile flukes, and the recommended drug in most countries, is not available in Botswana, most probably because of its high cost.

The low prevalence might also be attributed to the provision of better veterinary services to the farming community by private veterinary practitioners, whose numbers have increased in recent years, and who also provide advice to farmers on livestock management, which could reduce the prevalence of fasciolosis. In addition, owing to the large size of the bovine liver, it is also possible that the prevalence of fasciolosis was underestimated in the present study since some livers with partial infection could have been passed as fit for human consumption after trimming of the affected parts. Another possibility might be that farmers chose to send their healthiest animals to the main abattoirs and the less healthy or poor conditioned cattle to the local council abattoirs and meat inspection slabs situated around the country, where they expect less scrutiny during meat inspection of their animals.

The pattern of distribution of *F. gigantica* infections from this study showed that areas that receive higher rainfall had a higher prevalence than did cattle from the drier areas. A comparison of the findings between the two abattoirs showed that the Francistown abattoir, in the northern region, recorded a higher prevalence of the disease than the Lobatse abattoir, in the southern part of the country. The result is not surprising as the Francistown abattoir receives cattle from high rainfall areas in the north-east and central districts. The north-east district borders Matebeleland province in Zimbabwe, where a prevalence of 36.1%, based on an abattoir study, has been reported (Pfukenyi & Mukaratirwa 2004). This area receives an annual rainfall of more than 1000 mm, which provides good conditions for the survival of the intermediate host snail, *Lymnaea natalensis*.

The lowest prevalence of fasciolosis recorded at Lobatse abattoir in the southern part of the country could be attributed to the fact that the abattoir is largely supplied of cattle from Kgalagadi and Gantsi districts, where relatively dry conditions exist, which are unfavourable for the survival of the intermediate host snail. The mean annual rainfall for Lobatse is 550 mm, and it is even lower (250 mm – 300 mm) in Gantsi and Kgalagadi districts.

The observation from this study is, to some extent, in agreement with studies from other parts of Africa, where higher prevalence of fasciolosis in cattle was reported following periods of high rainfall and from areas with high rainfall than during drought periods and from areas with lower rainfall (Kithuka *et al*. 2002; Mungube *et al*. 2006; Pfukenyi & Mukaratirwa 2004). This is further evidence that rainfall has a direct influence on the occurrence of liver flukes (Kithuka *et al*. 2002), and the origin of cattle examined at a particular abattoir would be expected to have a strong influence on the prevalence of the disease (Phiri *et al*. 2005b). Fasciolosis is enzootic in areas with a mean annual rainfall of over 1000 mm where *L. natalensis* is widely distributed (Pfukenyi & Mukaratirwa 2004) (Table 2).

**TABLE 2:** Annual rainfall and temperature for the catchment areas of two export abattoirs in Botswana.

Abattoir	Catchment area	Range in annual rainfall (mm)	Range in temperature (°C)
Lobatse	Gaborone	400–850	18–28
	Kgatleng	400–850	18–30
	Kweneng	350–600	20–30
	Ngwaketse	350–600	18–30
	Tsabong	150–400	22–40
	Hukuntsi	150–400	22–40
	Gantsi	150–400	22–40
Francistown	Tutume	650–1200	22–35
	Masunga	650–1200	22–35
	Letlhakane	400–650	22–35
	Serowe	400–650	20–32
	Palapye	400–550	20–32
	Mahalapye	400–650	20–32
	Machaneng	400–650	20–32
	Selibephikwe	400–550	20–32
	Bobonong	400–550	20–32

The cool and humid climate in the central and north-east districts therefore probably provides the optimal conditions for the survival of the intermediate host snail and the liver fluke. *Lymnaea natalensis*, a freshwater snail, is common and widely distributed in tropical and subtropical Africa, including Botswana (Brown & Kristensen 1989; Seddon *et al*. 2010), and can tolerate a wide range of conditions, including changes affecting regional wetlands (Seddon *et al*. 2010). The snail is found in a great variety of habitats, including natural permanent water bodies, man-made dams, reservoirs, ponds and even cattle drinking troughs (Pfukenyi & Mukaratirwa 2004; Seddon *et al*. 2010). These water bodies increase the risk of acquisition of infection (Ogunrinade & Ogunrinade 1980). Therefore, the higher prevalence of the disease in cattle slaughtered at the Francistown abattoir was probably related to the presence of numerous streams and rivers in the north-east catchment area, as opposed to the ephemeral water system that is prevalent in most of the southern region catchment areas where the Lobatse abattoir is located. A similar study in Zambia by Phiri *et al*. (2005b) found a higher prevalence in areas prone to flooding.

There was no distinct seasonal pattern in liver condemnation rates and therefore the prevalence of fasciolosis. In contrast, elsewhere in Africa seasonal differences have been observed, with a high prevalence of the disease reported during the rainy or post-rainy season (Asanji & Williams 1984; Mzembe & Chaudhry 1981; Nonga *et al*. 2009; Pfukenyi & Mukaratirwa 2004; Phiri *et al*. 2005a). A study in Zimbabwe found that a snail population builds during the beginning of the dry season and then drops during the cold, dry months in winter, but again increases during the rainy season, with a concomitant peak in liver condemnations at the abattoirs (Pfukenyi & Mukaratirwa 2004). This is also a likely scenario in north-eastern Botswana.

## Conclusion

In conclusion, the findings of the present abattoir study have provided preliminary baseline data on the prevalence of bovine fasciolosis in Botswana. These results suggest that *F. gigantica* is not a major cause of liver condemnation in abattoirs in Botswana, and thus only low annual financial losses would have been incurred as a consequence of condemnation of *F. gigantica* infected livers during the 10-year period. There is a need, however, for a cross-sectional study of fasciolosis in cattle of all ages to determine the real situation in Botswana and to have a better understanding of the epidemiology of this important parasitic disease of ruminants to subsequently allow the design and implementation of appropriate control measures.
